# SNP rs2596542G>A in MICA is associated with risk of hepatocellular carcinoma: a meta-analysis

**DOI:** 10.1042/BSR20181400

**Published:** 2019-05-07

**Authors:** Haichuan Wang, Hui Cao, Zhong Xu, Dong Wang, Yong Zeng

**Affiliations:** 1 Department of Liver Surgery, Liver Transplantation Division, West China Hospital, Sichuan University, Chengdu, China; 2 Department of Oncology, Guizhou Provincial People’s Hospital, Guiyang, China; 3 Department of Gastroenterology, Guizhou Provincial People’s Hospital, Guiyang, China; 4 Department of Surgery, Transplant and Stem Cell Immunobiology (TSI-) Lab, University of California, San Francisco (UCSF), San Francisco, CA, U.S.A.

**Keywords:** HCC, MICA, rs2596542, single nucleotide polymorphisms

## Abstract

The association of major histocompatibility complex class I chain-related gene A (*MICA*) single nucleotide polymorphism (SNP) rs2596542G>A and hepatocellular carcinoma (HCC) has been broadly studied, with inconsistent results. Therefore, we conducted the current meta-analysis to better elucidate the roles of SNP rs2596542G>A in HCC. Eligible articles were searched in PubMed, CNKI, Wanfang, Embase, VIP, Web of Science, and CBM databases up to November 2018. Odds ratios (ORs) and 95% CIs were applied. A total of 11 articles, including 4528 HCC patients and 16,625 control subjects, were analyzed. Results revealed that rs2596542G>A was significantly associated with HCC in the heterozygote (G/A versus A/A, *P*=0.006, OR = 0.854; 95% CI: 0.763–0.956); and dominant (G/G + G/A versus A/A; *P*=0.021; OR = 0.796; 95% CI: 0.655–0.967) genetic models. Nevertheless, we also detected significant associations between rs2596542G>A and HCV-induced HCC. Additionally, according to our analyses, SNP rs2596542G>A was not correlated with HBV-induced HCC. In conclusion, our findings suggest that *MICA* SNP rs2596542G>A is associated with HCC susceptibility amongst the Asian, Caucasian, and African ethnicity in certain genetic models. Specifically, *MICA* SNP rs2396542G>A is associated with risk of HCV-induced HCC, not HBV-induced HCC.

## Introduction

Liver cancer is one of the leading causes of death worldwide responsible for nearly 746,000 deaths per year. It is reported to be the fifth most common cancer in men (7.5% of all cancers) and the ninth in women (3.4% of all cancers) occurred in 2012 [1]. Hepatocellular carcinoma (HCC), which is mainly associated with liver cirrhosis related to chronic hepatitis B virus (HBV) and/or hepatitis C virus (HCV) infection, accounts for the majority pathological type of primary liver cancer [2,3]. To date, there are very few effective treatments for HBV or HCV related-HCC because the pathogenic molecular- or genetic-based mechanisms are poorly understood.

HBV and HCV are discrepant viruses that target and persist in hepatocytes, leading to chronic liver diseases (CLD) and subsequent HCC [4,5]. CLD is a progressive liver disorder and consists of different liver pathologies, including hepatitis, fibrosis, cirrhosis, and eventually HCC [6,7]. Though both HBV and HCV can contribute to HCC, the role of them in HCC development is reported to be distinct [8]. The contribution of HBV and HCV as risk factors for HCC varies amongst different geographical areas and populations. Therefore, variation in genetic features has long been suspected and validated to contribute to the volatile risks for HCC amongst different populations. Preliminary evidence indicates that host genetic elements may conduce to predisposition of infection, occurrence of chronic hepatitis, progression of liver cirrhosis, and development of carcinoma [9,10]. Currently, one of the most extensively studied inherited genetic risk factors for virus-induced HCC are variants of the human major histocompatibility complex class I chain-related gene A (*MICA*) [11].

*MICA*, or Perth β block transcript 11 (PERB11), was first described by Bahram et al. in 1994 [12]. It is expressed on the cell surface as a ligand binding to the NKG2D type II integral membrane protein receptor as to modulates immune responses mediated by NK and T cells [13–15]. Functioning as a stress-induced antigen, *MICA* can be markedly up-regulated or expressed *de novo* when the organisms encounter stimulations from virus, bacteria, environment, and autoimmune conditions [16,17]. Considering that *MICA* plays an important role in immune activation and surveillance against infection and tumorigenesis, recent studies have mainly focussed on the association between *MICA* polymorphism and susceptibility to virus-associated cancers, including human papillomavirus-induced cervical cancer [18,19], HBV- or HCV-induced HCC [20–22], and Epstein–Barr virus-associated nasopharyngeal carcinoma [23,24].

The *MICA* single nucleotide polymorphism (SNP) rs2596542G>A is located in the promoter region of *MICA*, 4.7 kb upstream of *MICA* exon1, and 41.7 kb downstream of HLA-B. Therefore, SNP rs2596542G>A cannot change the *MICA* coding sequence [20]. However, it is crucial for initiating and promoting gene expression. Recently, *MICA* polymorphism rs2596542G>A has been identified to be associated with HCC. Tong et al. demonstrated that rs2596542G>A A polymorphism was associated with increased progression from HBV-induced cirrhosis to HCC in a case-controlled study in a Vietnamese cohort [21]. Conversely, Kumar et al. conducted a genome-wide association study (GWAS) amongst Japanese population showing that the SNP rs2596542G allele was considered to increase the risk of HBV-induced HCC and, in addition, that carriers of the rs2596542G allele presented higher serum MICA (sMICA) levels [25]. Interestingly, another similar study from the same Japanese group elucidated that SNP rs2596542A allele was a risk allele in HCV-associated HCC cases [20]. However, Lange et al. demonstrated that the A allele had a protective impact in HCV-induced HCC based on a Caucasian population sample [26]. Moreover, Burza et al. (2016) found no association between rs2596542G>A and HCC development in HCV-related HCC amongst a European population [27]. Altogether, these inconsistent results may be due to distinct epidemic genetic characteristics, limited sample sizes, little statistical power, and/or clinical heterogeneity. Therefore, we performed the current meta-analysis to shed light into the conflicting data situation regarding the association between *MICA* rs2596542G>A and the risk of HCC.

## Materials and methods

### Study selection

Two reviewers (Haichuan Wang and Hui Cao) performed searches in PubMed, CNKI, Wanfang, Embase, VIP, Web of Science, and CBM databases to ascertain studies which had presented the association between *MICA* polymorphisms and liver cancer, with the last updated search being conducted on November 2018. We conducted literature searches in PubMed database with the following strategy: (‘major histocompatibility complex class I-related chain A’ or ‘major histocompatibility complex class I chain-related gene A’ or ‘MHC class I polypeptide-related sequence A’ or *MICA* or MIC-A or PERB11.1) and (‘polymorphism*’ or SNP) and (liver or hepatic or hepatocellular) and (cancer or carcinoma or neoplasm or tumor). Searching strategies adopted in Embase and Web of Science database were adjusted based on these. There was no restriction for language. The following inclusion criteria were applied: (1) conducted as a case-control study on the association of MICA SNP and liver cancer risks; (2) OR and 95% CI can be counted based on the genotype frequencies provide in the study; (3) the genotype distributions in control groups should conform to the Hardy–Weinberg equilibrium (HWE); and (4) the study must be administrated in human samples. Exclusion criteria were as follows: (1) not carried out as a case-control study; (2) reviews, abstracts with no original data or overlapping studies with replicate data; and (3) unavailable genotype frequencies or other essential information in the study.

### Data extraction and quality assessment

Two reviewers (Haichuan Wang and Hui Cao) sorted out the essential information from every study on the basis of the inclusion and exclusion criteria. For missing information, we contacted with the authors for original information or raw data. If the reviewers encountered with any disagreements, they would discussed until reaching a consensus. Senior reviewers (Yong Zeng) eventually reviewed final results before moving to next step. The following information was extracted from the included studies: first author’s name and country, publication year, study subjects’ ethnicity, genotyping methods, control group number and case group number, allocations of alleles and genotypes in control subjects and HCC patients, and the control group *P* value for HWE (if applicable). To evaluate the quality of non-randomized studies regarding to comparability, selection, and exposure, we applied the quality score (QS) to estimate the qualification of included studies [28,29]. According to QS standards, studies were rated a score according to a quality assessment scale (Supplementary Table S1). Studies of high quality had to be scaled with a score of more than 9.

**Table 1:** Characteristics of studies included in the meta-analysis

First author	Year	Location	Genotyping method	Number of cases	Number of controls	Cases			Controls			P_HWE_ for control	QS^1^
						CC	CG	GG	CC	CG	GG		
Augello	2018	Italy	LGC Genomics	150	242	49	64	37	61	133	48	0.1113	7
Mohamed	2017	Egypt	TaqMan	47	47	6	32	9	19	23	5	0.6118	6
Huang	2017	Taiwan	TaqMan	58	647	27	26	5	318	256	73	0.0526	8
Hai	2017	Japan	TaqMan	142	575	57	58	27	246	269	60	0.2806	7
Li	2016	China	PCR-RFLP	120	124	48	57	15	65	52	7	0.4125	7
Tong	2013	Vietnam	TaqMan	163	417	62	74	27	169	196	52	0.6774	9
Lo	2013	Japan	GWAS^2^	1629	1043	587	777	265	459	450	134	0.1524	11
Lange	2013	Switzerland	PCR-AS^3^	64	1860	34	24	6	736	871	253	0.8535	9
Chen	2013	China	TaqMan	506	772	264	200	42	425	293	54	0.7183	10
Kumar	2012	Japan	GWAS	407	5657	205	165	37	2593	2375	689	0.0001	11
Kumar	2011	Japan	GWAS	1392	5483	505	660	227	2517	2304	662	0.0002	11

^1^QS, Quality score. ^2^GWAS, Genome wide association studies by Illumina Human Hap610-Quad or Human Hap550v3 or Invader assay. ^3^PCR-AS, Allele-specific PCR.

### Statistical analysis

Statistical analyses in the current study were performed with Stata 12.0 (Stata Corporation, College Station, TX, U.S.A.). To evaluate heterogeneity between studies, we adopted I^2^ statistic test and Q test. Between-study heterogeneity was significant if I^2^ was greater than 50% or the *P* value of Q test was less than 0.1. In this situation, we conducted analyses in a random-effect model (REM). Otherwise, if I^2^ was equal to or less than 50% or the *P* value of Q test was no more than 0.1, the study would be considered homogeneous. Therefore, a fixed-effect model (FEM) would be applied for analyses. Five genetic models (G/A versus A/A, G/G versus A/A, G versus A, G/G+G/A versus A/A, G/G versus G/A+A/A) were tested to explore any potential variations in the distribution of SNP rs2596542G>A amongst HCC cases and control subjects. Moreover, ORs and the corresponding 95% CIs were adopted to estimate associations of SNP rs2596542G>A with HCC, and statistically significance between HCC patients and control subjects was considered if the *P* value was equal to or less than 0.05. Furthermore, we conducted subgroup analyses based on distinct ethnicity and different virus types of study population to get more precise results. HWE in the control group was explored by using the χ^2^ test. Publication bias was assessed with Egger’s test and Beg’s funnel plots, *P* value of 0.05 or less was considered representative of statistically significant publication bias.

### Trail sequential analysis and false-positive report probability analyses

Trail sequential analysis (TSA) was performed by using TSA-Trial Sequential Analysis Viewer (version 0.9.5.10 β, Copenhagen Trial Unit, Copenhagen, Denmark) [30]. A level of significance of 5% for type I error and 30% for type II error was adopted. Then the required information size was generated, and TSA monitoring boundaries were built. The FPRP values at different prior probability levels for all significant findings were calculated as described [31]. We set 0.2 as FPRP threshold and assigned a prior probability of 0.1 to detect an OR of 0.67/1.5 (for protective/risk effects) for an association with genotypes under investigation. A FPRP value <0.2 denoted a noteworthy association.

## Results

### Study characteristics

**Figure 1:**
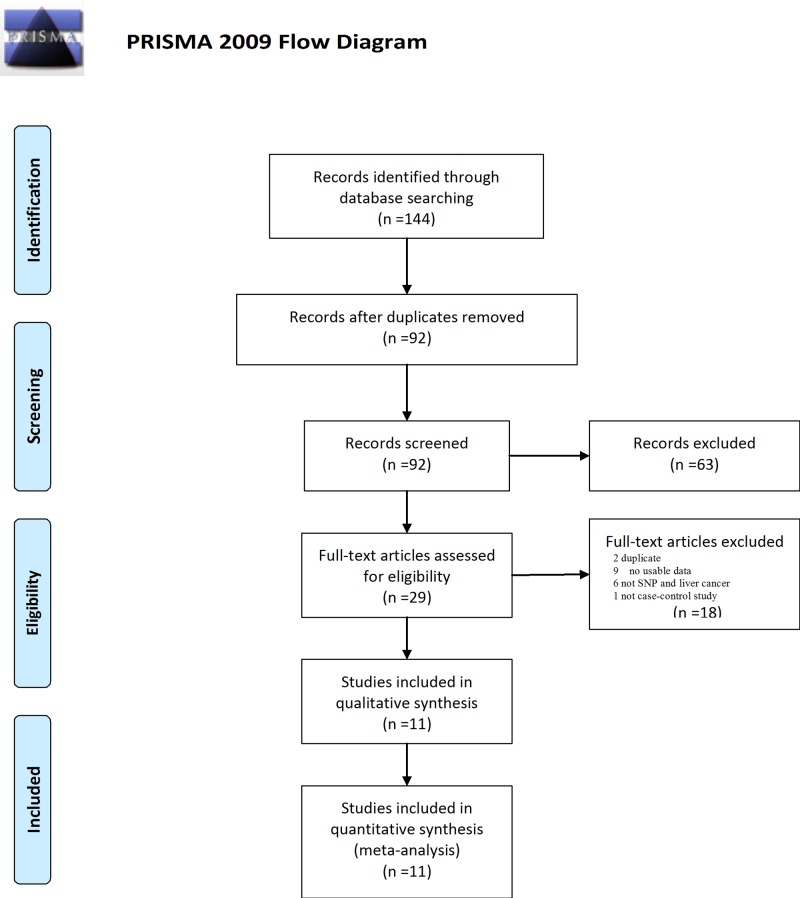
Flowchart of study selection for current study

The literature search generated 144 potential results for selection. After removing duplicated articles, 92 records were included for further evaluation. By reading titles and abstracts, 63 records were excluded for either irrelevant studies or duplicated studies. Altogether, 29 articles were screened for full-text assessment. Amongst these, two articles were excluded for duplication, nine articles were excluded for no usable data, six articles were excluded for not SNP and liver cancer studies, and one article was exclude for not case-control study. Therefore, a total of 11 studies containing 4528 HCC patients and 16,625 controls were finally included (Figure 1), 7/11 were about the association of rs2596542G>A and HCV-induced HCC, 3/11 were about the rs2596542G>A and HBV-induced HCC [20–22,25,26,32–37]. Included studies were all published between 2011 and 2018. Of these, eight studies were amongst Asian ethnicity, two studies were amongst Caucasian, and one study was amongst African. Notably, 3/11 genome wide association studies were also included for further analyzing. We also evaluated the HWE test for the control group in each study. Results indicated that *P* value of χ^2^ test was greater than 0.1 in each study except for two GWAS. However, they were also included for further analysis because a HWE *P*<1.0 × 10^−6^ for controls was applied as standard SNP quality control in these two studies [20]. Characteristics of studies investigating are summarized in Table 1.

### Association of rs2596542G>A and HCC

**Figure 2:**
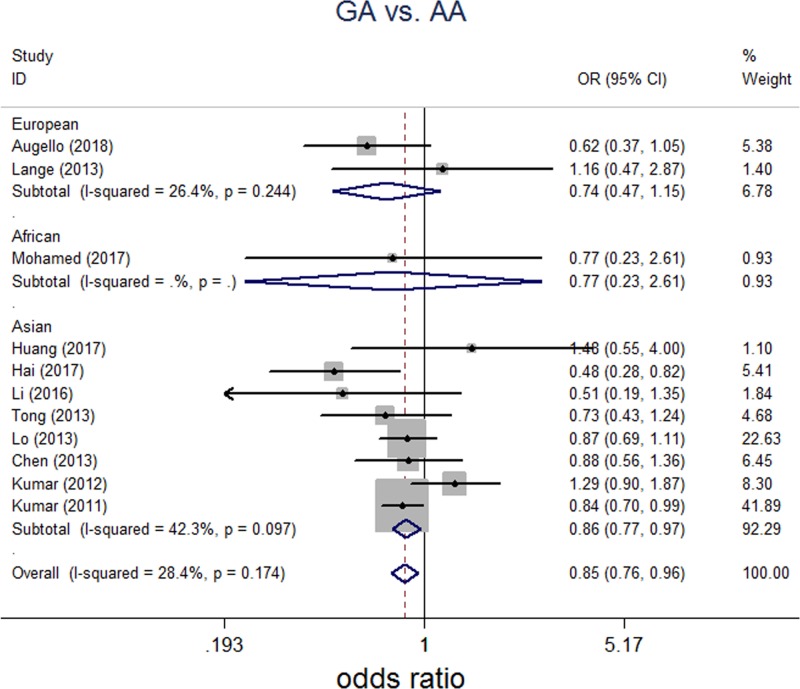
Forest plot on association between rs2596542G>A polymorphism and HCC risk (G/A versus A/A) Fix-effect pooled OR = 0.854, 95% CI: 0.763–0.956, *P*=0.006, I^2^ = 28.4%, *P* for the heterogeneity 0.174.

**Figure 3:**
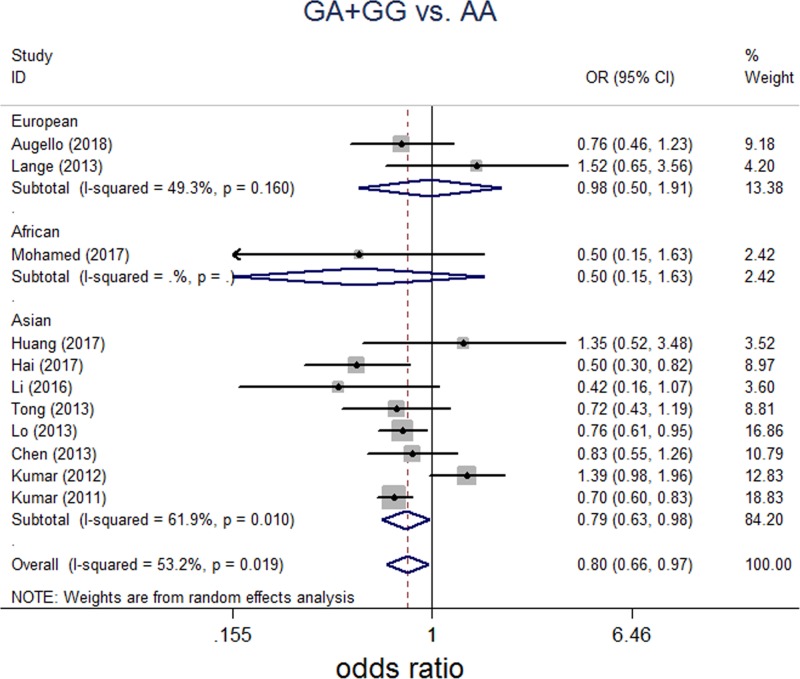
Forest plot on association between rs2596542G>A polymorphism and HCC risk (G/A+G/G versus A/A) Random-effect pooled OR = 0.796, 95% CI: 0.655–0.967, *P*=0.021; I^2^ = 53.2%, *P* for the heterogeneity 0.019.

A total of 11 independent studies consisting of 4528 HCC patients and 16,625 healthy controls were included for analyzing association of rs2596542G>A and susceptibility of HCC. All genetic models were tested to explore any potential differences in genotypic and allelic frequencies regarding to SNP rs2596542G>A amongst HCC cases and control. For G/A versus A/A, between-study heterogeneities were trivial, so FEM was applied for analyses. For G/G versus A/A, G versus A, G/G+G/A versus A/A, and G/G versus G/A+A/A, analyses were performed with REM because of significant between-study heterogeneities. Overall, significant association between rs2596542G>A and HCC was found in G/A versus A/A (*P*=0.006, OR = 0.854; 95% CI: 0.763–0.956, Figure 2), G/G+G/A versus A/A (*P*=0.021; OR = 0.796; 95% CI: 0.655–0.967, Figure 3). However, there was no significant association between rs2596542G>A and HCC in G/G versus A/A, G versus A, or G/G versus G/A+A/A models (Table 2).

**Table 2:** Meta-analysis results of association between rs2596542G>A and HCC

Sample	Comparison	Number of studies	Test of heterogeneity		Meta-analysis results		Comparison model
			P(Q-test)	I^2^	OR (95% CI)	*P*	
Overall	GG vs AA	11	0.000	72.50%	0.765 (0.584–1.003)	0.053	REM
	**GA vs AA**	**11**	**0.174**	**28.40%**	**0.854 (0.763–0.956)**	**0.006**	**FEM**
	**GA+GG vs AA**	**11**	**0.019**	**53.20%**	**0.796 (0.655–0.967)**	**0.021**	**REM**
	GG vs GA+AA	11	0.000	80.10%	0.884 (0.726–1.076)	0.219	REM
	G vs A	11	0.000	78.80%	0.882 (0.768–1.014)	0.077	REM
**Subgroup by pathogen**							
HBV	GG vs AA	3	0.031	71.20%	0.967 (0.601–1.555)	0.890	REM
	GA vs AA	3	0.166	44.30%	1.019 (0.797–1.304)	0.880	FEM
	GA+GG vs AA	3	0.055	65.60%	0.965 (0.638–1.460)	0.867	REM
	GG vs GA+AA	3	0.117	53.50%	1.008 (0.813–1.250)	0.944	REM
	G vs A	3	0.026	72.50%	0.991 (0.800–1.227)	0.931	REM
HCV	**GG vs AA**	**7**	**0.017**	**61.20%**	**0.707 (0.530–0.942)**	**0.018**	**REM**
	**GA vs AA**	**7**	**0.305**	**16.40%**	**0.821 (0.723–0.934)**	**0.003**	**FEM**
	**GA+GG vs AA**	**7**	**0.278**	**19.80%**	**0.730 (0.647–0.824)**	**0.000**	**FEM**
	GG vs GA+AA	7	0.000	79.50%	0.867 (0.669–1.122)	0.278	REM
	**G vs A**	**7**	**0.002**	**70.70%**	**0.854 (0.732–0.998)**	**0.047**	**REM**
**Subgroup by ethnicity**							
European	GG vs AA	2	0.240	27.50%	1.287 (0.806–2.056)	0.291	FEM
	GA vs AA	2	0.244	26.40%	0.735 (0.471–1.146)	0.174	FEM
	GA+GG vs AA	2	0.160	49.30%	0.920 (0.610–1.389)	0.693	FEM
	**GG vs GA+AA**	**2**	**0.590**	**0.00%**	**1.562 (1.120–2.181)**	**0.009**	**FEM**
	G vs A	2	0.153	51.00%	1.227 (0.870–1.729)	0.244	REM
Asian	**GG vs AA**	**8**	**0.001**	**72.60%**	**0.729 (0.554–0.959)**	**0.024**	**REM**
	GA vs AA	8	0.097	42.30%	0.852 (0.708–1.027)	0.093	REM
	**GA+GG vs AA**	**8**	**0.010**	**61.90%**	**0.785 (0.630–0.978)**	**0.031**	**REM**
	**GG vs GA+AA**	**8**	**0.000**	**75.20%**	**0.833 (0.698–0.994)**	**0.043**	**REM**
	**G vs A**	**8**	**0.000**	**78.20%**	**0.855 (0.744–0.982)**	**0.027**	**REM**
**Subgroup by quality score**							
>9	GG vs AA	4	0.000	85.80%	0.798 (0.550–1.158)	0.235	REM
	GA vs AA	4	0.209	33.90%	0.898 (0.792–1.017)	0.090	FEM
	GA+GG vs AA	4	0.007	75.40%	0.862 (0.659–1.127)	0.277	REM
	GG vs GA+AA	4	0.000	88.40%	0.839 (0.653–1.077)	0.169	REM
	G vs A	4	0.000	89.80%	0.882 (0.724–1.075)	0.214	REM
≤9	GG vs AA	7	0.019	60.50%	0.723 (0.456–1.144)	0.166	REM
	**GA vs AA**	**7**	**0.434**	**0.00%**	**0.688 (0.532–0.890)**	**0.004**	**FEM**
	**GA+GG vs AA**	**7**	**0.205**	**29.20%**	**0.717 (0.564–0.912)**	**0.007**	**FEM**
	GG vs GA+AA	7	0.003	69.90%	0.909 (0.648–1.275)	0.579	REM
	G vs A	7	0.011	64.00%	0.879 (0.706–1.095)	0.249	REM

### Association of rs2596542G>A and HBV- or HCV-induced HCC

**Figure 4:**
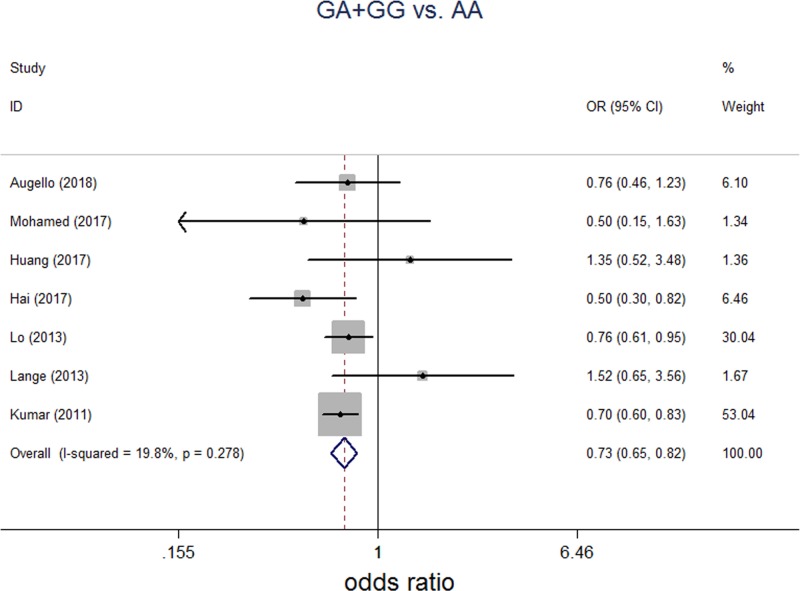
Forest plot on association between rs2596542G>A polymorphism and HCV-induced HCC risk (G/A+G/G versus A/A) Fixed effect pooled OR = 0.730, 95% CI: 0.647–0.824; *P*<0.001; I^2^ = 19.80%, *P* for the heterogeneity 0.278.

To further elucidate the varieties between HBV-induced HCC and HCV-induced HCC regarding to SNP rs2596542G>A, we did subgroup analysis based on hepatitis group. In HBV group, a total of three studies including 1076 HBV-induced HCC patients and 6846 healthy controls were included. For the evaluation of the association between SNP rs2596542G>A and HBV-induced HCC, we assessed genotype and allele frequencies in HBV-induced HCC group and control group amongst all five genetic models. For G/A versus A/A, FEM was applied for no significant between-study heterogeneity. For G/G versus A/A, G versus A, G/G+G/A versus A/A, and G/G versus G/A+A/A, REM was applied for significant between-study heterogeneities. No significant association of SNP rs2596542G>A and HBV-induced HCC was found in G/A versus A/A, G/G versus A/A, G versus A, G/G+G/A versus A/A, and G/G versus G/A+A/A models (Table 2). In HCV group, a total of seven studies including 3482 HCV-induced patients and 9879 healthy controls were included. For the evaluation of the association between SNP rs2596542G>A and HCV-induced HCC, we assessed genotype and allele frequencies in HCV-induced HCC group and control group amongst all five genetic models. For G/A versus A/A and G/G+G/A versus A/A, FEM was applied for no significant between-study heterogeneities. For G/G versus A/A, G versus A and G/G versus G/A+A/A, REM was applied for obvious between-study heterogeneities. A significant association of SNP rs2596542G>A and HCV-induced HCC was found in G/G versus A/A (*P*=0.018; OR = 0.707; 95% CI: 0.530–0.942), G/A versus A/A (*P*=0.003; OR = 0.821; 95% CI: 0.723–0.934), G versus A (*P*=0.047; OR = 0.854; 95% CI: 0.732–0.998) and G/G+G/A versus A/A (*P*=0.000; OR = 0.730; 95% CI: 0.647–0.824, Figure 4). However, there was no significant association between SNP rs296542 and HCV-induced HCC in G/G versus G/A+A/A model (Table 2).

### Subgroup analysis based on ethnicity

In certain genetic models for association between SNP rs2596542G>A and HCC, between-study heterogeneities were detected to be significant. Considering that ethnic background is one of the major sources of heterogeneity in studies relating to genetic predisposition, we therefore conducted subgroup analysis by separately analyzing studies with same ethnic background. Two studies consisting of 214 HCC cases and 2102 controls were included in Caucasian group while eight studies compromising 4417 HCC cases and 14,718 controls were enrolled in Asian group. For Caucasian group, the striking between-study heterogeneities were minimized to a great extent. All genetic models except for G versus A were applied to FEM. A significant association of SNP rs2596542G>A and HCC was found in G/G versus G/A+A/A (*P*=0.009, OR = 1.562; 95% CI: 1.120–2.181). However, the striking between-study heterogeneities remained unchanged in the Asian subgroup. Therefore, REM was applied to all genetic models. Significant association were found in G/G versus A/A (*P*=0.024; OR = 0.729; 95% CI: 0.554–0.959), G versus A (*P*=0.027; OR = 0.855; 95% CI: 0.744–0.982), G/G+G/A versus A/A (*P*=0.031; OR = 0.785; 95% CI: 0.630–0.978), and G/G versus G/A+A/A (*P*=0.043; OR = 0.833; 95% CI: 0.698–0.994). However, no significant association between SNP rs2596542G>A and HCC were found in G/A versus A/A model (Table 2).

### Subgroup analysis based on QS

To further explore other potential sources of heterogeneity, we stratified all the studies into high score group (QS>9) and low score group (QS≤9). A significantly decreased risk of HCC was observed in low score group (Table 2): G/A versus A/A (*P*=0.004; OR = 0.688; 95% CI: 0.532–0.890), G/G+G/A versus A/A (*P*=0.007; OR = 0.717; 95% CI: 0.564–0.912). However, no significant association between rs2596542G>A and HCC was observed in high score group (Table 2). Besides, after stratification by QS, SNP rs2596542G>A showed a significant association of HCV-induced HCC in high score group with a trend of decreased OR values (Supplementary Table S2). Furthermore, after stratification by QS, SNP rs2596542G>A showed a significant association amongst Asian cohort in low score group with a trend of decreased OR values. However, no significant association was found amongst Asian cohort in high score group (Supplementary Table S3).

**Table 3:** False positive report probability values for association between rs2596542G>A and HCC.

Variables	OR (95% CI)	*P*^1^	Power^2^	Prior probability^3^				
				0.25	0.1	0.01	0.001	0.0001
**GA vs AA**								
All	0.854 (0.763–0.956)	0.006	1.000	**0.01801**	**0.05214**	0.37700	0.85928	0.98390
HCV	0.821 (0.723–0.934)	0.003	0.999	**0.00810**	**0.02391**	0.21225	0.73110	0.96456
QS≤9	0.688 (0.532–0.890)	0.004	0.595	0.02176	0.06256	0.42331	0.88105	0.98669
**GG vs GA+AA**								
European	1.562 (1.120–2.181)	0.009	0.406	**0.06126**	**0.16372**	0.68289	0.95601	0.99542
Asian	0.833 (0.698–0.994)	0.043	0.993	**0.11421**	0.27891	0.80970	0.97724	0.99768
**GA+GG vs AA**								
All	0.796 (0.655–0.967)	0.021	0.889	**0.07675**	**0.19960**	0.73285	0.96513	0.99640
HCV	0.730 (0.647–0.824)	0.000	0.929	**0.00000**	**0.00000**	**0.00004**	**0.00038**	**0.00379**
Asian	0.785 (0.630–0.978)	0.031	0.927	**0.09087**	0.23069	0.76737	0.97083	0.99701
QS≤9	0.717 (0.564–0.912)	0.007	0.723	**0.02710**	**0.07713**	0.47899	0.90270	0.98935
**G vs A**								
HCV	0.854 (0.732–0.998)	0.047	0.999	**0.12396**	0.29800	0.82362	0.97922	0.99788
Asian	0.855 (0.744–0.982)	0.027	1.000	**0.07396**	**0.19330**	0.72496	0.96376	0.99626

^1^Chi-square test was adopted to calculate the genotype frequency distributions.

^2^Statistical power was calculated using the number of observations in the subgroup and the OR and *P* values in this table.

^3^Values for prior probability in bold implies noteworthiness at 0.2 level.

### Publication bias

**Figure 5:**
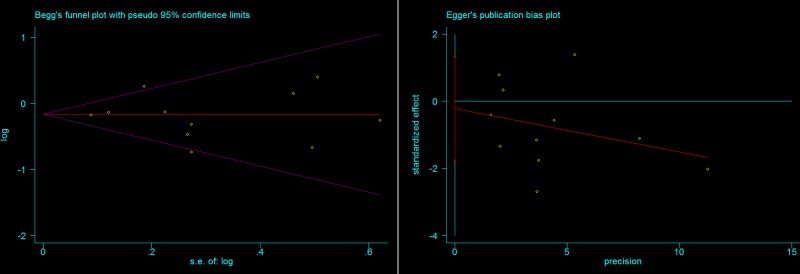
Begg’s funnel plot (*P*=0.983) and Egger’s funnel plot for publication bias (*P*=0.709) in G/G+G/A versus A/A model

We tested potential publication bias by adopting Begg’s funnel plots and Egger’s funnel plots. No apparent asymmetry of funnel plots was visually inspected. In G/G+G/A versus A/A model, *P* value for Begg’s funnel plot and Eggers funnel plots is 0.938 and 0.709 (Figure 5), indicating that there was no significant publication bias.

### TSA and FPRP analyses

**Figure 6:**
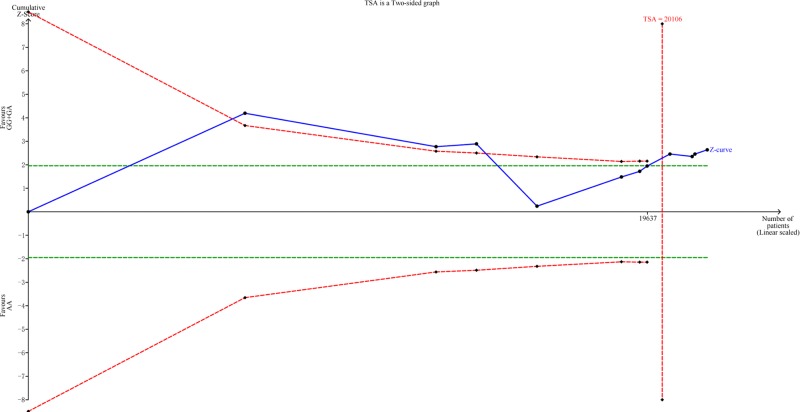
TSA between rs2596542G>A polymorphism and HCC risk (G/A+G/G versus A/A)

We conducted TSA for the dominant model (G/G+G/A versus A/A) to narrow down the random errors and strengthen the robustness of our conclusions (Figure 6). Results indicated that the cumulative z-curve crossed the trial sequential monitoring boundary (type I error 5%, Z score = 1.96) before reaching the required information size (TSA = 20,106). Therefore, the cumulative evidence is sufficient, and no further evidence is needed to verify the conclusions.

We finally calculated the FPRP values for all observed significant findings. With the assumption of a prior probability of 0.2, the FPRP values and statistic power for significant findings at different prior probability levels are shown in Table 3. Take dominant model (G/G+G/A versus A/A), for an example, for a prior probability of 0.1, if the OR for specific genotype was 0.67/1.50 (protection/risk), with statistic power of 0.889, the FPRP value was 0.1996 for an association of SNP rs2596542G>A and HCC risk in all individuals. Positive association between SNP rs2596542G>A and HCC risk observed in subgroups of HCV and QS≤9 were considered noteworthy findings because their probability to be a false positive result was lower than 0.2. However, we observed a greater FPRP value for the significant association between SNP rs2596542G>A in Asian population and HCC risk, indicating that some possible bias existed due to reduced sample size in this subgroup, which need further validation in lager studies.

## Discussion

HCC has always been one of the most widespread primary tumors and accounts for almost 90% of primary liver cancers. Generally, risk factors of HCC include excessive consumption of alcohol, HBV, and HCV infection and aflatoxin B. Its heterogeneity and geographic variability has been tightly related to different HBV or HCV susceptible factors worldwide, leading to different epidemic features of HBV- or HCV-associated HCC. Although the mechanisms of HBV- or HCV-related HCC have been widely studied, the exact mechanisms remain to be further explored. With respect to genetic risk factors, there are many studies focus on the polymorphisms in the *MICA*. Kumar et al. first found a previously unidentified locus in the 5′ flanking region of *MICA* on 6p21.33 (rs2596542G>A) to be strongly associated with HCV-induced HCC in a GWAS [20]. The rs2596542G>A at restriction site has an absolute linkage within the *MICA* promoter region and may alter the binding of stress inducible transcription factors. Tong et al. [14] hypothesized that the SNP rs2596542G>A could affect the expression of *MICA* or initiate pathways related with tumor development. Therefore, studies on the relationship of HCC risk and *MICA* SNP rs2596542G>A emerged with inconclusive results from different study cohort. Thus, to get a more conclusive and convincing results, we conducted this comprehensive meta-analysis.

This meta-analysis, including 11 case-control studies from 11 research articles, investigated SNP rs2596542G>A amongst Asian, Caucasian, and African people. We found that the rs2596542G>A polymorphism was associated with a decreased risk of HCC in G/A versus A/A and G/A+G/G versus A/A from a total of 4528 cases and 16,625 controls. These results indicate that GA heterozygotes or G allele carriers in MICA SNP rs2596542G>A are potentially protected amongst all the population. Consistent with our finding, Li et al. [35] reported that A/A genotype increased the onset risk of HCC amongst Chinese population in a case-control study consisting of 120 HCC patients and 124 healthy controls. Considering that the between group heterogeneity was high in the overall analysis, we performed a subgroup analysis in terms of different ethnicity. As it is expected, meta-analysis amongst Asian group showed even more significant association between rs2596542G>A and HCC. Similarly, G allele and G/G genotype could diminish the risk of HCC development amongst Asian population. Strikingly, we found an increased risk amongst G/G patients compared with G/A+A/A patients amongst Caucasian people. This indicates that homozygous G/G genotype is associated with an increased risk of HCC in Caucasian people. Similarly, Augello et al. [32] reported that rs2596542G>A G/G genotype carriers had a higher HCC risk and a significantly higher level of s*MICA* in a study amongst Sicilian population. Moreover, they even demonstrated that rs2596542G>A G/G homozygote showed a higher HCC risk in association with age following multivariate adjustment. These are also consistent with Lange et al.’s study, which elucidated that minor allele A of rs2596542G>A, had a protective impact on HCC amongst a Swiss cohort study. However, study amongst African population is limited. Mobhanded et al. [33] reported that rs2596542G>A G allele was observed to contribute to decreased risk of HCC amongst Egyptian population. Taken together, we claim that there are ethnicity-related variables in the recurrence of rs2596542G>A polymorphisms risk allele and minor allele A of rs2596542G>A and this may potentially protect Caucasian population from HCC while increase the risk of HCC amongst Asian and African population. More studies are needed for further validating our results.

Generally, while HBV is the major etiological factor in high incidence HCC areas, HCV is the main causative agent in low incidence HCC areas, such as western Europe and North America. Therefore, we also conducted subgroup analysis of HBV-induced HCC and HCV-induced HCC. No significant association between polymorphism rs2596542G>A and HBV-induced HCC was found. This result is contradictory to a GWAS conducted by Kumar et al. [25] amongst a Japanese population. Moreover, Tong et al. [21] also found a significant association between rs2596542G>A and HBV-induced HCC and the minor A allele contributed to an increased risk of HCC. However, our result is consistent with the conclusion of Chen et al.’s [37] study based on a Chinese Han population. The inconsistent results between studies may be caused by variable sample size and different genetic background. However, considering that our results are based on highly eligible studies with relatively high quality and the large sample size of the present analysis, the results of the current study may be more convincing than previous single cohort studies. As for HCV-induced HCC group, we found significant association between SNP rs2596542G>A and HCV-induced HCC. Based on the meta-analysis results, the major allele G in rs2596542G>A contributes to the decreased risk of HCV-induced HCC. Interestingly, this consequence is in opposite with two studies amongst Caucasian population while it is consistent with studies amongst Asian population. Altogether, we claim that SNP rs2596542G>A is associated with HCV-induced HCC.

Clinically, several studies have indicated that sMICA can be used as a prognostic marker for various malignant diseases [38,39]. sMICA levels are also reported to be associated with the progression of HBV-induced HCC [25]. Interestingly, sMICA levels are tightly correlated with *MICA* SNP rs2596542G>A according to previous studies [40]. Therefore, variations in *MICA* SNP rs2596542G>A can be used a genetic indicator for HCC progression to provide new thoughts into genetic therapy of malignant diseases.

There are still some disadvantages in our meta-analysis. First, we currently only included the published studies in the selected electronic databases. Therefore, some relevant published data or unpublished studies with raw data might be missed. It might cause bias of our results. Second, we did not stratify data into other subgroups on the basis of other potentially factors including age, gender, and study location. Third, correlation between genetic factors and environmental factors relating to polymorphisms should be discussed more thoroughly. Fourth, some included studies in this meta-analysis have small sample size, which shall influence publication bias. Fifth, some other polymorphisms in *MICA* gene are not discussed in the current study because of limited reachable raw data. It might not completely elucidate the functional role of *MICA* in HCC development. Therefore, a new meta-analysis shall be administrated with more available high-quality studies in future.

Despite the weaknesses, we have improved our study identification and data selection process by double-checked policy and minimized potential bias of publication bias and sensitivity. Furthermore, we conducted FPRP analysis to investigate potential false positive report probability of the included studies. Most significant results were noteworthy at a FPRP level of 0.2 except for results from Asian subgroup, which indicates that more evidence need to be collected. Moreover, TSA results indicate that evidences for all individuals under dominant model are enough and no more evidence is needed to ensure the robustness of our results. Thus, we believe the current results are reliable.

In conclusion, the current meta-analysis indicates that *MICA* rs2596542G>A polymorphism is associated with susceptibility to HCV-induced HCC, suggesting that *MICA* polymorphism plays an important role in HCV-associated liver cancer progression. Large scale, well-designed case-control studies are still warranted to validate our results.

## Supporting information

**Supplemental Table S1:** Score of quality assessment

**Supplemental Table S2:** Subgroup analysis results by quality score of association between *rs2596542G > A* and HCV-induced HCC

**Supplemental Table S3:** Subgroup analysis results by quality score of association between *rs2596542G > A* and HCC among Asian cohort

## Abbreviations

CLD: chronic liver disease
FEM: fixed-effect model
FPRP: false-positive report probability
GWAS: genome-wide association study
HBV: hepatitis B virus
HCC: hepatocellular carcinoma
HCV: hepatitis C virus
HWE: Hardy–Weinberg equilibrium
OR: odds ratio
QS: quality score
REM: random-effect model
SNP: single nucleotide polymorphism
TSA: trail sequential analysis
