# Supplementary material for: SNP rs2596542G>A in MICA is associated with risk of hepatocellular carcinoma: a meta-analysis
Source: Biosci Rep. 2019 May 7;39(5):BSR20181400. doi: 10.1042/BSR20181400 (PMC6504665; doi:10.1042/BSR20181400)
Supplement: Supplementary file 1 [file bsr20181400_Supp1.pdf]

**Supplementary Table 1**. **Score of quality assessment**

| Criteria* | Score |
|---|---|
| **Representativeness of case** | |
| Selected from population cancer registry | 2 |
| Selected from hospital | 1 |
| No method of selection described | 0 |
| **Representativeness of control** | |
| Population-based | 3 |
| Blood donors | 2 |
| Hospital-based | 1 |
| Not described | 0 |
| **Ascertainment of HCC case** | |
| Histopathologic confirmation or CT/MRI* | 2 |
| by patient medical record | 1 |
| Not described | 0 |
| **Control selection** | |
| Controls matched with cases by age and sex | 2 |
| Controls matched with cases only by age or by sex | 1 |
| Not matched or not descried | 0 |
| **Genotyping examination** | |
| Genotyping done blindly and quality control | 2 |
| Only genotyping done blindly or quality control | 1 |
| Unblinded and without quality control | 0 |
| **HWE** | |
| HWE in the control group | 1 |
| HWD in the control group or not mentioned | 0 |
| **Total sample size** | |
| > 1000 | 3 |
| 501 - 1000 | 2 |
| 201 - 500 | 1 |
| ≤ 200 | 0 |

*Slightly modified according to the NCCN Clinical Practice Guidelines in oncology (NCCN Guidelines). Hepatobiliary Cancers. Version 1. 2018.

**Study quality is defined as low if QS⩽9 or high if QS＞9.

**Supplementary Table 2** Subgroup analysis results by quality score of association between *rs2596542G>A* and HCV-induced HCC

| Sample | Comparison | Number of Studies | Test of Heterogeneity | | Meta-analysis Results | | Comparison Model |
|---|---|---|---|---|---|---|---|
| | | | P(Q-test) | $I^2$ | OR (95% CI) | P | |
| High* | **GG vs. AA** | **2** | **0.513** | **0.00%** | **0.608(0.526, 0.702)** | **0.000** | **FEM** |
| | **GA vs. AA** | **2** | **0.769** | **0.00%** | **0.849(0.737, 0.977)** | **0.022** | **FEM** |
| | **GA+GG vs. AA** | **2** | **0.601** | **0.00%** | **0.724(0.634, 0.827)** | **0.000** | **FEM** |
| | **GG vs. GA+AA** | **2** | **0.517** | **0.00%** | **0.687(0.624, 0.757)** | **0.000** | **FEM** |
| | **G vs. A** | **2** | **0.446** | **0.00%** | **0.756(0.706, 0.810)** | **0.000** | **FEM** |
| Low* | GG vs. AA | 5 | 0.014 | 67.90% | 0.818(0.436, 1.536) | 0.532 | REM |
| | **GA vs. AA** | **5** | **0.237** | **27.70%** | **0.698(0.512, 0.950)** | **0.022** | **FEM** |
| | GA+GG vs. AA | 5 | 0.123 | 44.90% | 0.760(0.570, 1.013) | 0.061 | FEM |
| | GG vs. GA+AA | 5 | 0.003 | 74.50% | 0.972(0.611, 1.545) | 0.903 | REM |
| | G vs. A | 5 | 0.011 | 69.50% | 0.934(0.694, 1.257) | 0.653 | REM |

**Abbreviations:** FEM, Fixed-effect Model; REM, Random-effect Model.

*High: QS＞9.   **Low: QS≤9.

**Supplementary Table 3** Subgroup analysis results by quality score of association between *rs2596542G>A* and HCC among Asian cohort

| Sample | Comparison | Number of Studies | Test of Heterogeneity | | Meta-analysis Results | | Comparison Model |
|---|---|---|---|---|---|---|---|
| | | | P(Q-test) | $I^2$ | OR (95% CI) | P | |
| High* | GG vs. AA | 4 | 0.000 | 85.80% | 0.798(0.550, 1.158) | 0.235 | REM |
| | GA vs. AA | 4 | 0.209 | 33.90% | 0.898(0.792, 1.017) | 0.090 | FEM |
| | GA+GG vs. AA | 4 | 0.007 | 75.40% | 0.862(0.659, 1.127) | 0.277 | REM |
| | GG vs. GA+AA | 4 | 0.000 | 88.40% | 0.839(0.653, 1.077) | 0.169 | REM |
| | G vs. A | 4 | 0.000 | 89.80% | 0.882(0.724, 1.075) | 0.214 | REM |
| Low** | **GG vs. AA** | **4** | **0.265** | **24.40%** | **0.616(0.443, 0.856)** | **0.004** | **FEM** |
| | **GA vs. AA** | **4** | **0.229** | **30.50%** | **0.657(0.474, 0.912)** | **0.012** | **FEM** |
| | **GA+GG vs. AA** | **4** | **0.226** | **31.10%** | **0.636(0.468, 0.865)** | **0.004** | **FEM** |
| | GG vs. GA+AA | 4 | 0.589 | 0.00% | 0.838(0.676, 1.038) | 0.106 | FEM |
| | **G vs. A** | **4** | **0.419** | **0.00%** | **0.814(0.697, 0.951)** | **0.010** | **FEM** |

**Abbreviations:** FEM, Fixed-effect Model; REM, Random-effect Model.

*High: QS＞9.   **Low: QS≤9.
